# Purification of PaTx-II from the Venom of the Australian King Brown Snake and Characterization of Its Antimicrobial and Wound Healing Activities

**DOI:** 10.3390/ijms24054359

**Published:** 2023-02-22

**Authors:** Ramar Perumal Samy, Stephen P. Mackessy, Alagarmalai Jeyasankar, Mano Ranjana Ponraj, Octavio Luiz Franco, Matthew A. Cooper, Matheswaran Kandasamy, Tapan Kumar Mohanta, Jebasingh Bhagavathsingh, Sakthivel Vaiyapuri

**Affiliations:** 1 Department of Anatomy, Yong Loo Lin School of Medicine, National University of Singapore, NUHS, MD10, 4 Medical Drive, Singapore 117594, Singapore; 2 School of Biological Sciences, Campus Box 92, College of Natural and Health Sciences, University of Northern Colorado, 501 20th St., Greeley, CO 80639, USA; 3 Department of Zoology, Government Arts College (Autonomous), Coimbatore 641018, Tamil Nadu, India; 4 Medicinal Organic Chemistry Laboratory, Department of Applied Chemistry, Karunya Institute of Technology and Sciences, Coimbatore 641114, Tamil Nadu, India; 5 Centro de Análises Proteômicas e Bioquímicas, Programa de Pós-Graduação em Ciências Genômicas e Biotecnologia, UCB, Brasilia 71966-700, DF, Brazil; 6 Sinova Biotech, Programa de Pós-Graduação em Biotecnologia, Universidade Católica Dom Bosco, Campo Grande 79117-900, MS, Brazil; 7 Institute for Molecular Bioscience, The University of Queensland, St. Lucia, QLD 4072, Australia; 8 Radcliffee Department of Medicine, University of Oxford, Oxford OX3 9DU, UK; 9 Natural and Medical Sciences Research Center, University of Nizwa, Nizwa 611, Oman; 10 HPLC Mass Facility, Department of Applied Chemistry, Karunya Institute of Technology and Sciences, Coimbatore 641114, Tamil Nadu, India; 11 School of Pharmacy, University of Reading, Reading RG6 6UB, UK

**Keywords:** wound, antimicrobial, antibiotic, PaTx-II, fusidic acid ointment, Australian king brown snake venom, *Pseudechis australis*

## Abstract

Infections caused by multi-drug-resistant (MDR) bacteria are a global threat to human health. As venoms are the source of biochemically diverse bioactive proteins and peptides, we investigated the antimicrobial activity and murine skin infection model-based wound healing efficacy of a 13 kDa protein. The active component PaTx-II was isolated from the venom of *Pseudechis australis* (Australian King Brown or Mulga Snake). PaTx-II inhibited the growth of Gram-positive bacteria in vitro, with moderate potency (MICs of 25 µM) observed against *S. aureus*, *E. aerogenes,* and *P. vulgaris*. The antibiotic activity of PaTx-II was associated with the disruption of membrane integrity, pore formation, and lysis of bacterial cells, as evidenced by scanning and transmission microscopy. However, these effects were not observed with mammalian cells, and PaTx-II exhibited minimal cytotoxicity (CC_50_ > 1000 µM) toward skin/lung cells. Antimicrobial efficacy was then determined using a murine model of *S. aureus* skin infection. Topical application of PaTx-II (0.5 mg/kg) cleared *S. aureus* with concomitant increased vascularization and re-epithelialization, promoting wound healing. As small proteins and peptides can possess immunomodulatory effects to enhance microbial clearance, cytokines and collagen from the wound tissue samples were analyzed by immunoblots and immunoassays. The amounts of type I collagen in PaTx-II-treated sites were elevated compared to the vehicle controls, suggesting a potential role for collagen in facilitating the maturation of the dermal matrix during wound healing. Levels of the proinflammatory cytokines interleukin-1β (IL-1β), interleukin-6 (IL-6) and tumor necrosis factor-α (TNF-α), cyclooxygenase-2 (COX-2) and interleukin-10 (IL-10), factors known to promote neovascularization, were substantially reduced by PaTx-II treatment. Further studies that characterize the contributions towards efficacy imparted by in vitro antimicrobial and immunomodulatory activity with PaTx-II are warranted.

## 1. Introduction

*Staphylococcus aureus* is a major cause of infections in many communities as well as healthcare centers, and many strains show resistance to multiple antimicrobial agents [1]. It is estimated that *S. aureus* causes approximately 120,000 bloodstream infections globally in each year [2], and more than 90% of *S. aureus* strains are resistant to beta-lactam antibiotics [3]. In addition, S. aureus is the second most frequent pathogen found in neonatal bacteremia in the UK, resulting in significant morbidity as well as mortality rates of around 20–35% [4]. Further, a multi-center hospital sampling-based study showed an average methicillin-resistant *S. aureus* (MRSA) rate of 35.3% in all clinical isolates of S. aureus and 46.7% among *S. aureus* isolated from patients in intensive care units in Singapore [5]. Recent infections of >1.28 million people, resulting in 18,285 deaths every year in the USA alone, are mainly due to infections caused by multi-drug resistant bacteria (MDR) [6,7,8]. In Australia, >175,000 predicted annual cases of healthcare-acquired infection occurred after admittance to hospitals, and notably, the 854,289 bed days of prolonged stay for treatment of these symptoms results in severe economic impacts [9]. Gram-positive bacteria are responsible for 30–60% of invasive surgical infections [10], and they can exacerbate suppurative diseases, toxic shock syndrome [11], psoriasis [12], pneumonia, *Staphylococcal* scaled skin syndrome (SSSS), and cystic fibrosis [13,14]. Severe wound infections due to *S. aureus* can result in a very poor quality of life, amputation, and loss of life [15]. After injury or infection of a wound, immune cells, particularly resident macrophages (Mφ), are triggered by pro-inflammatory mediators IL-1α, IL-1β, IL-6, tumor necrosis factor-α (TNFα), cyclooxygenase-2 (COX-2) and phospho-p65, which trigger transcription factor NF-κB [16,17]. Transforming growth factor β (TGF-β), a cytokine, also plays a vital role in inflammation and angiogenesis [18].

In the face of these health threats, antibiotic discovery and development efforts with new entity approvals are only at early-stage trials with fewer new classes of antibiotics [19]. Novel approaches are needed to discover and develop better efficacious chemotherapeutics for treating *S. aureus* and other resistant bacterial infections. Snake venom phospholipases A_2_ [20], and associated antimicrobial peptides, have shown activity against *S. aureus* and *P. aeruginosa* [21], and interestingly, they have also been demonstrated to activate innate immune signaling [22,23]. Antimicrobial peptides (AMPs) have also been found in psoriatic lesions, where they inhibit the growth of bacteria [24,25]. Snake venoms also possess potent bioactivities with potential applications in the areas of medical, biotechnological and pharmaceutical importance [26,27,28]. Snake venoms often contain a large number of phospholipases A_2_ (PLA_2_) enzymes. Notably, a group of PLA_2_ homologues present in snake venoms, known as lysine-49 (Lys49) PLA_2_s, display bactericidal activity [29,30,31,32] and wound healing properties [33,34,35]. Several other snake venom proteins and peptides have previously been proved with antibacterial activity against Gram-positive and Gram-negative bacteria [36]. L-amino acid oxidase (LAAO), from the venom of *Cerastes vipera*, exerted bactericidal effects against *S. aureus* and *E. coli* (MIC of 20 µg/mL) [37], and a synthetic peptide (Pc-CoaTxII) derived from a Lys49 phospholipase A_2_ of *Crotalus oreganus abyssus* venom reproduced the antibacterial effects of the original toxin on multi-resistant clinical isolates [38]. Cardiotoxin 1 (CTX-1)-derived peptides from *Naja atra* (Chinese cobra) venom displayed broad-spectrum antimicrobial effects on fungi species such as *Candida albicans, C. glabrata*, and *Malassezia pachydermatis* (MBC of 50–6.3 µg/mL), *Mycobaterium smegmatis/M. fortuitum*, etc.

The active molecules in snake venom have great potential as drugs for treating human diseases, notably Naja cardiotoxin peptide-3 (NCP-3), exhibited anti-viral activity on bovine Herpesvirus 1 (BoHV1). Furthermore, it shows bactericidal action against methicillin resistant *S. aureus* (MRSA)*, E. coli*, and *P. aeruginosa* at high salt concentrations [39]. Antimicrobial peptides (AMP) derived from svPLA_2_ can also interact with teichoic acids, lipoteichoic acids/peptidoglycan of *S. aureus*, and lipopolysaccharide (LPS), specifically the lipid A component of Gram-negative bacteria, causing membrane permeabilization as well as potent bactericidal effects [40,41]. A small peptide also influences wound closure in an oral rat model [42]. It is reported that there is a high level of type I collagen mRNA as well as hydroxyl proline expression in the epidermis of burn wounds in transgenic mice after 12 days [43]. In yet another application of snake venom toxins, results clearly showed that the crotoxin fraction 1 (F1 CTX), a heterodimeric PLA_2,_ isolated from the venom of *Crotalus durissus terrificus*, had toxic effects on human immortal cell lines (including pancreatic, esophagus, cervical and glioma cancer cells) at concentrations up to 30 µg/mL. F1 CTX showed greater cytotoxic/pro-apoptotic effects in the glioma, pancreatic and cervical cancer cells, but less toxic effects on esophagus cells. Interestingly, F1 CTX failed to affect the viability of normal cells in vitro [44].

To our knowledge, only two reports in the literature describe the antibacterial screening of *Pseudechis australis* (Australian king brown) or Mulga snake (Msn) venom [45,46]. The earlier study indicated only the pilot screening and isolation of the high molecular weight proteins. “Two antibacterial proteins such as L-amino acid oxidase (molecular weights 142,000 of LAO1/142,000 of LAO2) isolated from the venom of Australian elapid, *Pseudechis australis* (AKB or Msn) to homogeneity. The antibacterial activity was correlated with enzymatic activity/eliminated with catalase. However, in vitro antibacterial effects of LAO1 and LAO2 were 70 as well as 17.5 times more effective on a molar basis when compared to tetracycline, a drug choice for *Aeromonas* infections in human, reptiles/amphibians etc” [47]. MS is the largest and among the most dangerous/wide-ranging venomous snakes in Australia and New Guinea [48]. Herein, we report the antibacterial effects of *P. australis* toxin-II (PaTx-II) isolated from the venom and tested against clinical pathogens under in vitro and in vivo settings.

## 2. Results

### 2.1. Purification of PLA_2_s from the Venom of P. australis

**Figure 1:**
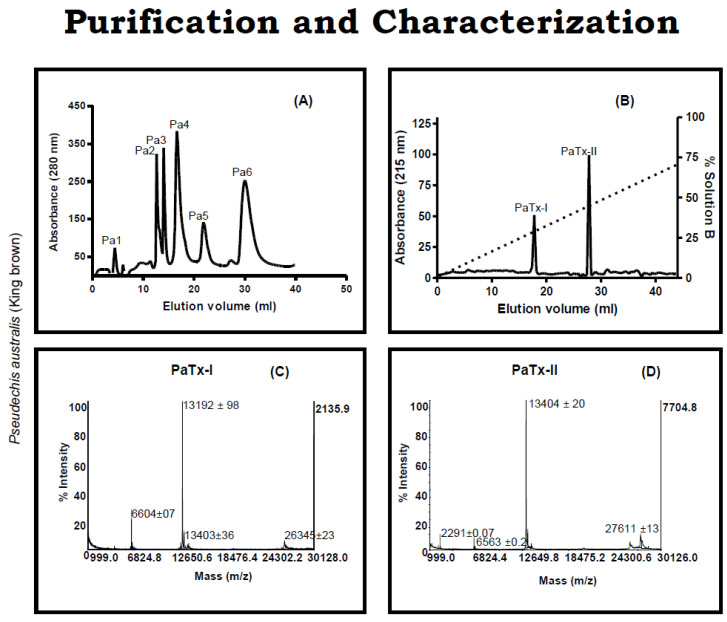
Purification and characterization of snake venom proteins. (**A**) Six fractions (Pa1–Pa6) were separated from the crude venom of *P. australis* (king brown) by a Superdex G-75 column equilibrated with 50 mM Tris-HCL buffer (pH 7.4). (**B**) The fractions (Pa4) containing antibacterial and PLA_2_ activities were further purified by reverse-phase (RP)-HPLC on C18 and resolved into six peaks (Pa-F1 to Pa-F6). The most active peak (Pa-F2) was further separated on a C8 column, producing two pure proteins, PaTx-I and PaTx-II. (**C**,**D**) Molecular weights of the svPLA_2_ proteins (PaTx-I and PaTx-II) were determined using MALDI-TOF/MS.

The venom of *P. australis* was separated into six major protein fractions (Pa1-Pa6) using a Superdex G-75 column. Fraction Pa4 had strong PLA_2_ activity (Table 1), and therefore this fraction was further purified using a C18 column, which resolved into six peaks (Figure 1A). From the latter, the most active fraction (Pa-F2) was further purified on a C8 column and resolved into two isolated proteins designated as PaTx-I and PaTx-II (Figure 1B). The apparent molecular masses of PaTx-I (13,192 ± 98) (Figure 1C) and PaTx-II (13,404 ± 20) (Figure 1D) were determined by Matrix Assisted Laser Desorption Ionization-Time of Flight (MALDI-TOF). The alignment of N-terminal sequences of PaTx-I and PaTx-II show that they are highly homologous.

### 2.2. Antimicrobial Properties of PaTx-II

**Figure 2:**
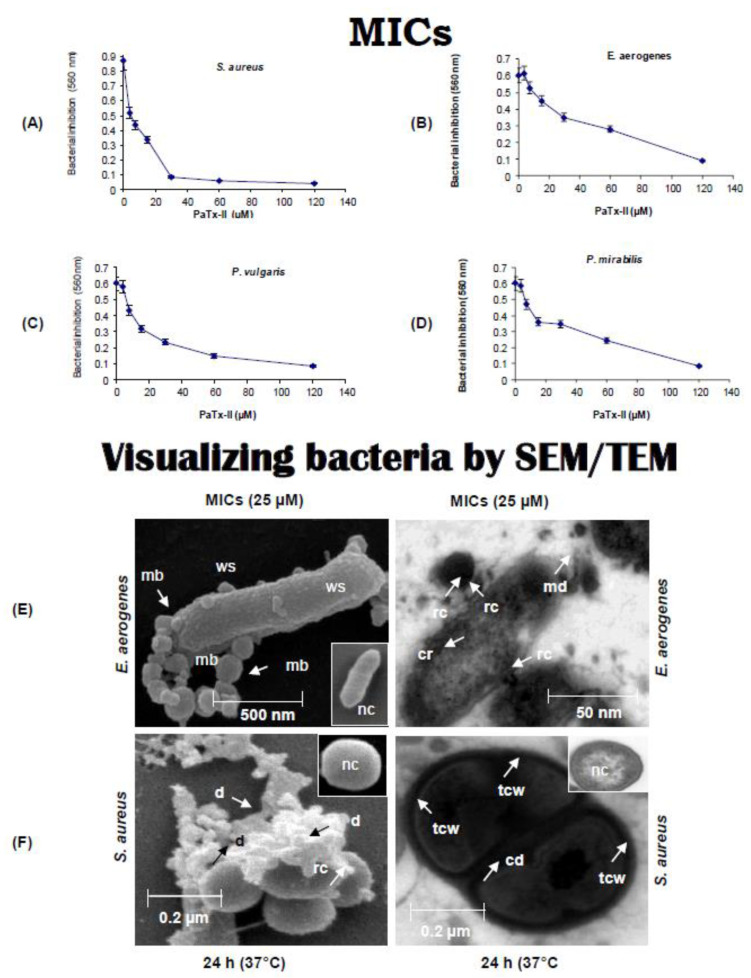
Antimicrobial potency of PaTx-II was tested against bacteria for 24 h at 37 °C. (**A**–**D**) Minimum inhibitory concentrations (MICs) were determined by standard procedures (see Methods). The inhibitory effect of PaTx-II was shown against *S. aureus* (MICs 25 µM), *P. vulgaris* (MICs 28 µM), *P. mirabilis* (MICs 35 µM) and *E. aerogenes* (MICs 25 µM); svPLA_2_ shows the most potent inhibitory effect at the lowest dilutions. (**E**,**F**) Ultrastructural (scanning and transmission electron microscope) micrographs reveal that the PaTx-II-treated (MIC, 25 µM) bacteria show membrane blebs (mb), cell roughening (cr), debris (d), release of cellular contents (rc), wrinkled surfaces (ws), thickened cell walls (tcw), membrane damage (md) and cell death (cd) at the septal region after 24 h at 37 °C. Untreated normal cells (nc, insets) show none of these features.

PaTx-II displayed a modest antibacterial activity against *S. aureus* and *E. aerogenes* compared to antibiotics (FAO and chloramphenicol) at 120 µM (Table 1). Growth inhibitory activities of PaTx-I and PaTx-II were evaluated against several bacteria, including *S. aureus*, *E. aerogenes*, *P. vulgaris*, and *P. mirabilis* (Figure 2A–D). *Pseudomonas aeruginosa* failed to show any susceptibility to PaTx-II (Table 1), although PaTx-II was more effective as an antibacterial agent than PaTx-I. The areas where PaTx-II affects *S. aureus* appear to be lamellar mesosome-like structures at the bacterial membrane, and striking electron-dense deposits were affixed to the outer membrane (Figure 2E,F).

### 2.3. Effect of PaTx-II on Human Cell Lines

**Figure 3:**
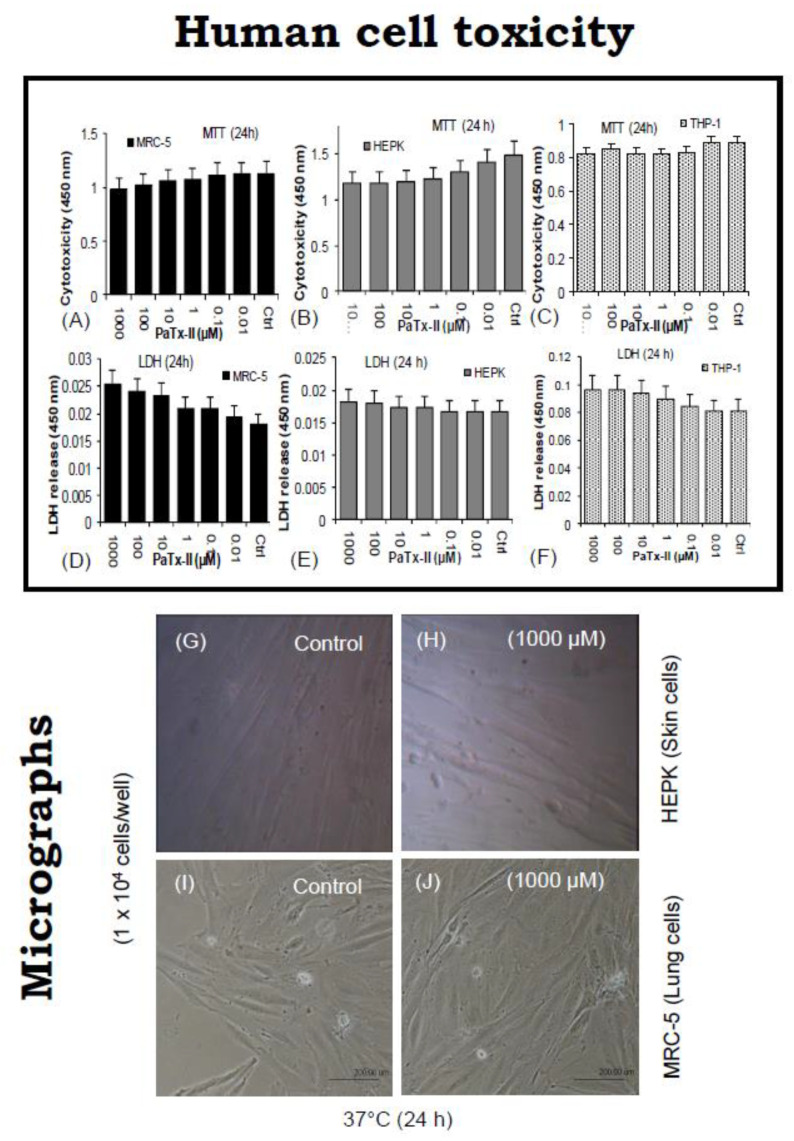
Cytotoxicity of the PaTx-II protein on cultured lung (MRC-5), skin fibroblasts (HEPK), and human macrophage (THP-1) cells incubated with 0.1–1000 µM doses for 24 h. (**A**–**C**) Cytotoxicity was measured by the MTT assay, and results reveal that PaTx-II did not induce dose-dependent toxic effects, even at higher doses (1000 µM). The untreated cells are included as controls. (**D**–**F**) Lactate dehydrogenase (LDH) released from cells. PaTx-II did not induce cell death or lytic effects at all assayed concentrations. Data represent the mean ± standard deviation (SD) of the three independent replicates (n = 3). (**G**,**H**) Light micrographs showing the cellular morphology in the presence of PaTx-II. (**G**,**I**) Control cells show normal morphology, (**H**,**J**) PaTx-II-treated skin and lung fibroblasts did not induce any morphological changes after 24 h.

To determine the cytotoxic effects of PaTx-II on human cells, the effects on human skin (HEPK), lung fibroblast (MRC-5), and macrophage (THP-1) cell growth were determined ex vitro (Figure 3A–C). There was no reduction of cell proliferation up to 1000 µM of PaTx-II compared to the controls. Interestingly, there was a significant enhancement of cell numbers observed after the treatment of PaTx-II (*p* < 0.05). On the other hand, cell proliferation was further increased up to a 1000 µM dose (*p* < 0.01) after treatment with PaTx-II. In Figure 3D–F, PaTx-II did not induce any cell death or lytic effects at any concentrations, and no morphological changes were observed in the light micrographs (Figure 3G–J).

### 2.4. Wound Healing by PaTx-II PLA_2_

**Figure 4:**
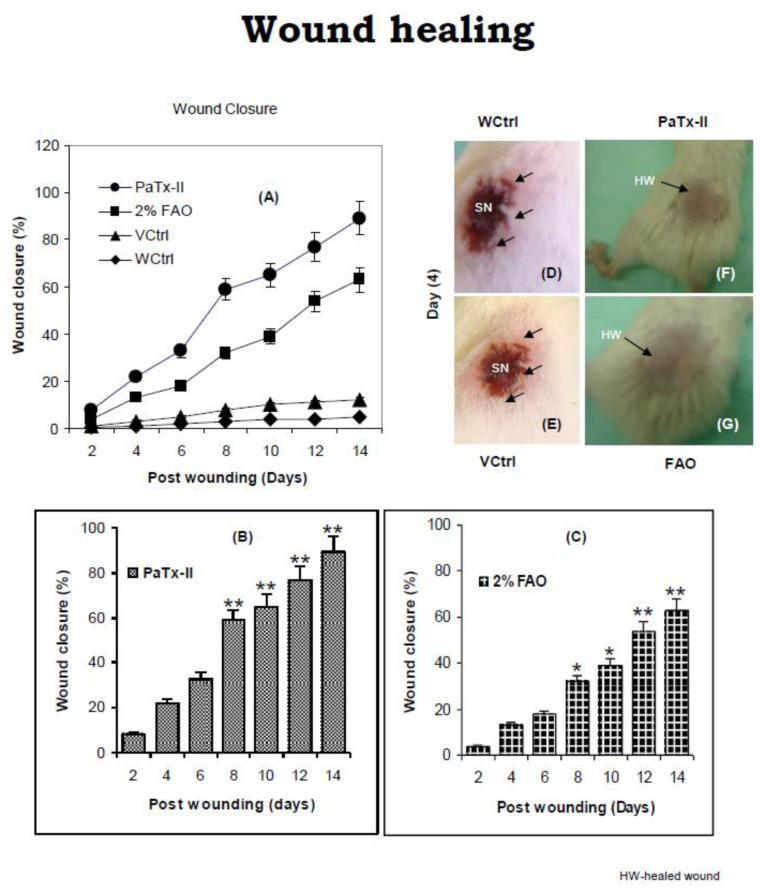
Wound healing effects of proteins in the mouse model. (**A**) *S. aureus*-infected wound treated with PaTx-II or fusidic acid ointment (FAO) shows enhanced healing when applied to mice at 0.5 mg/kg. Wound contraction was compared between the wound control (WCtrl) and vehicle control (VCtrl) mice. Control mice show severe necrosis (SN) and muscle damage after 4 days. (**B**) Percent reduction of wound after treatment with PaTx-II was analyzed between 2 and 14 days. The progress of the wound repair processes is indicated in days. (**C**) Post-treatment wound closure progression after treatment with 2% FAO (5 mice per group). HW represents the healing wound. Wound photographs: (**D**) Day 4, WCtrl. (**E**) Day 4, VCtrl. (**F**,**G**) Action of PaTx-II or FAO-completely healed wounds. (**A**–**C**) Data are the mean ± SD of 5 animals per group (n = 5) and represent three independent experiments. Statistical comparisons were performed with Prism 4.0 Software (Graph-Pad), using a two-tailed Student’s *t*-test for the comparison of two data sets, and ANOVA for multiple comparisons. Statistically significant differences are shown, with *p* values of * *p* < 0.05 and ** *p* < 0.01.

The PaTx-II treatment enhanced wound healing and clearing of *S. aureus* infection in mice to a greater extent than fusidic acid ointment (FAO), or Fucidin, compared to the untreated control group (Figure 4A). To monitor the changes in wound areas, the following PaTx-II and 2% of FAO applications were taken as indices of wound closure. PaTx-II-treated mice show significant improvements in wound healing with reduced inflammation, and in the clearing of *S. aureus*. The treated wound areas in PaTx-II mice were more rapidly reduced compared to control mice, and by day 8, wound areas in PaTx-II-treated mice show significantly greater closure (60%) than FAO treated mice (40%) (Figure 4B,C). There was increased inflammation, redness, and maximal scarring due to the bacterial infection found in the wound in control mice on day 4, whereas the vehicle control animals show less redness and scarring (Figure 4D,E). Overall, wounds on the PaTx-II-treated mice exhibited no redness and minimal scarring versus FAO-treated mice (Figure 4F,G). These observations demonstrate that wound closure, complete clearance of *S. aureus*, and eventual healing were accelerated in the presence of PaTx-II. The wound was completely healed after 14 days.

### 2.5. Clinicopathological Examination of Wound Tissue

**Figure 5:**
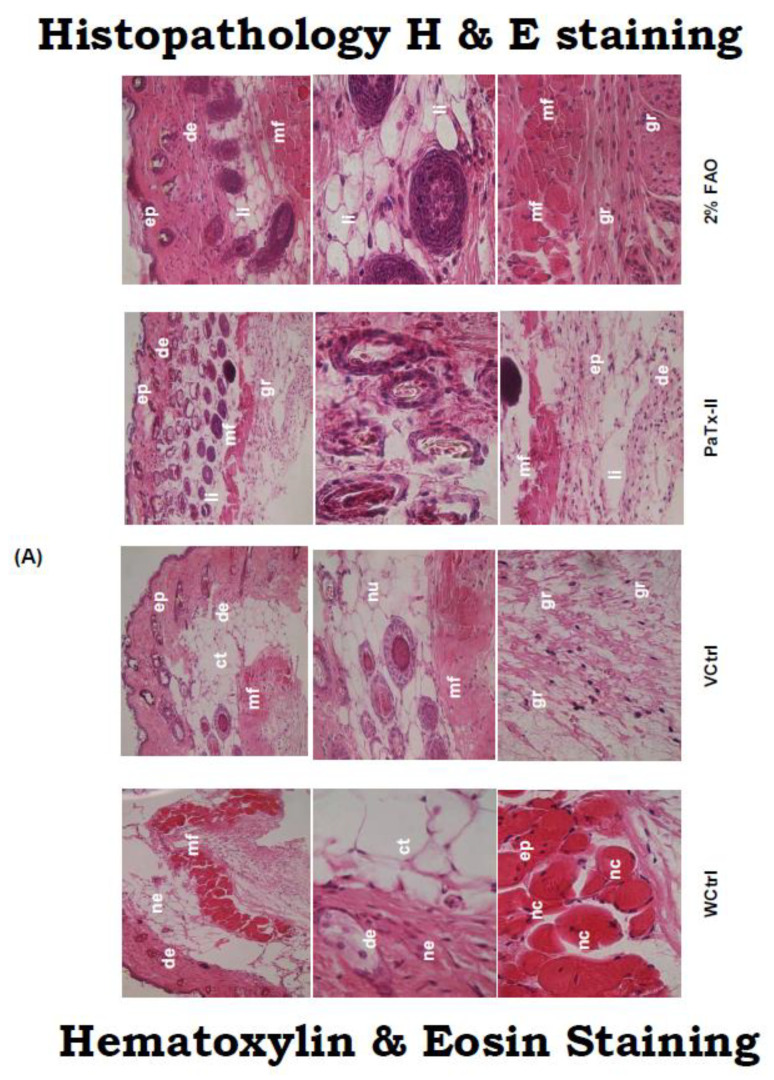

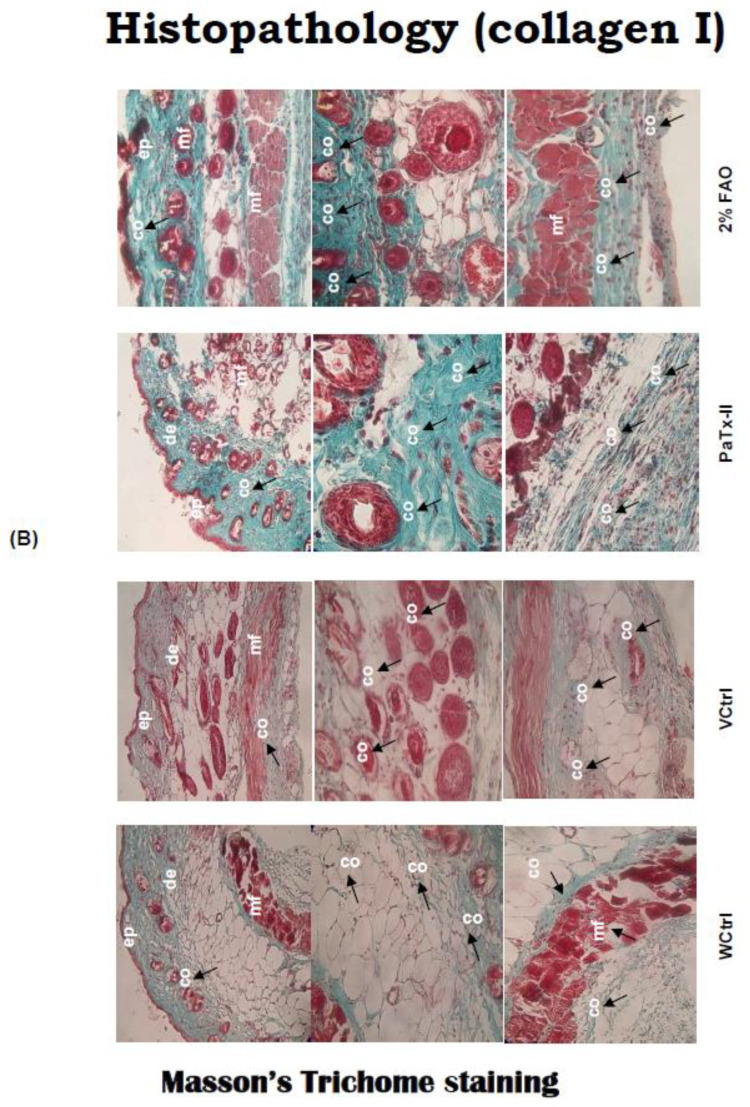

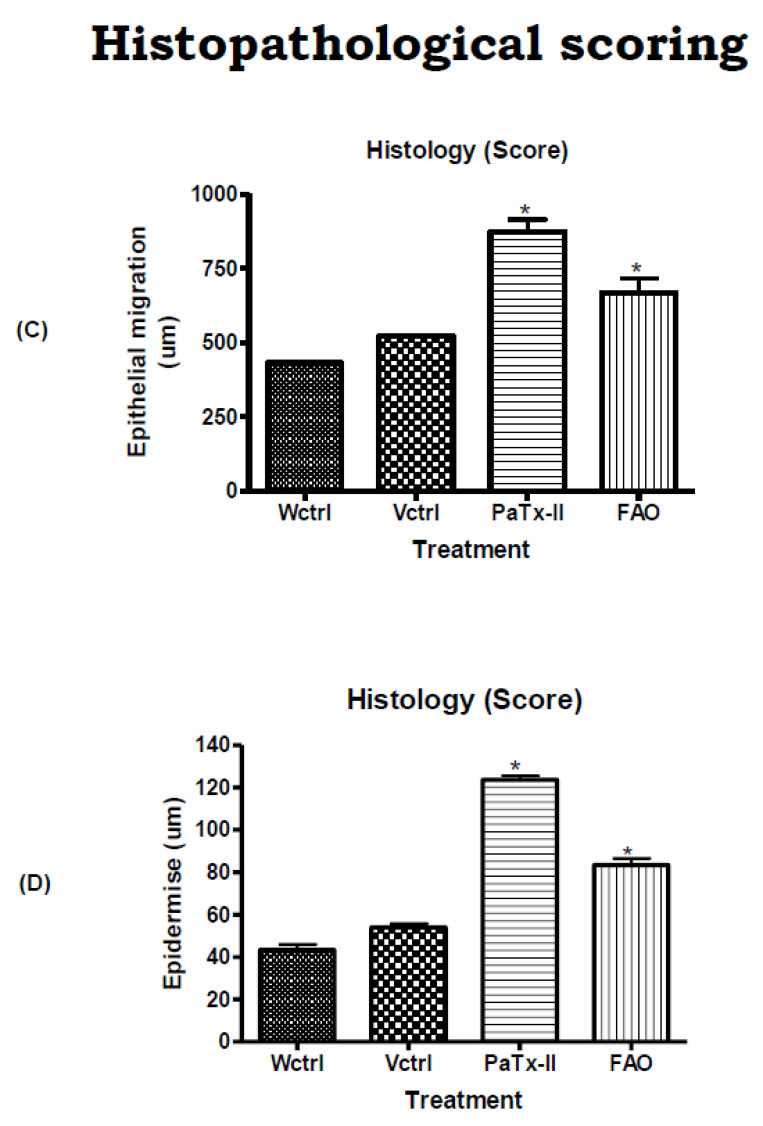
(**A**) Pathological assessment of wounds before and after treatment was evaluated under the light microscope. Group (I): WCtrl mice show redness and inflammation. Group (II): Among VCtrl mice, there was necrosis (nc) and abundant inflammatory infiltrates, changes in the muscle fibers (mf) and almost complete destruction of the mf cells after injury on day 14. Group (III): PaTx-II locally applied to mice exerted significant healing and clearing of bacteria after 14 days. Group (IV): Two percent FAO-treated mice healed more slowly than the PaTx-II treatment group. Abbreviations: ep-epidermis, de-dermis, li-leukocyte infiltration, mf-muscle fiber, nu-neutrophils, gr-granules, ne-necrosis (original magnification: ×20–×100). (**B**) MT staining detection of collagen deposition in skin tissues of different wound healing groups before and after treatment. G (I)—WCtrl and G(II)—VCtrl collagen was not detected in either group up 14 days post-wounding. G (III): PaTx-II-treated mice show abundant expression of collagen at 14 days after local application. G (IV): Two precent FAO-treated mice also equally expressed collagen for complete healing (original magnification: ×20-×100). (**C**) Epithelial migration from the wound edge increases the thickness of the epithelial layer 14 days post wounding. (**D**) Epidermal migration from both edges of the wound was measured with an ocular micrometer. Three measurements were used for each wound (n = 3); values are expressed as mean of epithelial migration ± SD thickness of epithelium. Both treatments show a significant difference (* *p* < 0.05) from controls (WCtrl and VCtrl), and there was also a significant difference (* *p* < 0.05) between PaTx-II and FAO-treated mice. ‘co’ and ‘ct’ on images represent controls.

Inflammatory cells and abundant neutrophilic infiltrates were found in the undamaged control and vehicle-treated mice. Sections of hematoxylin and eosin (H&E) stained tissues of PaTx-II-treated and control mice were observed for the epithelial regeneration, infiltration of cells, the density of late-formed vessels, and the organization of the granulation tissue. It is noteworthy to report the significant observations, including enhanced numbers of granules and epidermal keratinocytes, which lead to accelerated wound healing, were prominent in PaTx-II-treated mice after 14 days. The wounds were comprised of granulation tissue containing many long, spindle-shaped fibroblasts with noticeable nuclei and several capillaries. Inflammatory cells such as neutrophils, lymphocytes, and mononuclear cells were also observed in treatment groups versus controls. Wound structure was noticeable regarding collagenization and the arrangement of collagen fibers, similar to skin. In addition, PaTx-II-treated wounds not only contained more cells and vessels within the granulating tissues but also promoted epithelial migration compared to VCtrl (Figure 5A–C).

### 2.6. Biological Functions of PaTx-II

#### Masson’s Trichrome (MT) Staining for Collagen Expression

Collagen I expression was examined by MT staining and was evident throughout the wound site of the skin. PaTx-II-treated wounds had the bundles of type I collagen lying parallel to the surface between the fibrinous exudates and granulating tissue (Figure 5D). Collagen expression was also higher in the 2% FAO-treated group versus controls. However, the collagen-I staining was not detected in either WCtrl or VCtrl controls.

### 2.7. Clearance of Bacteria

**Figure 6:**
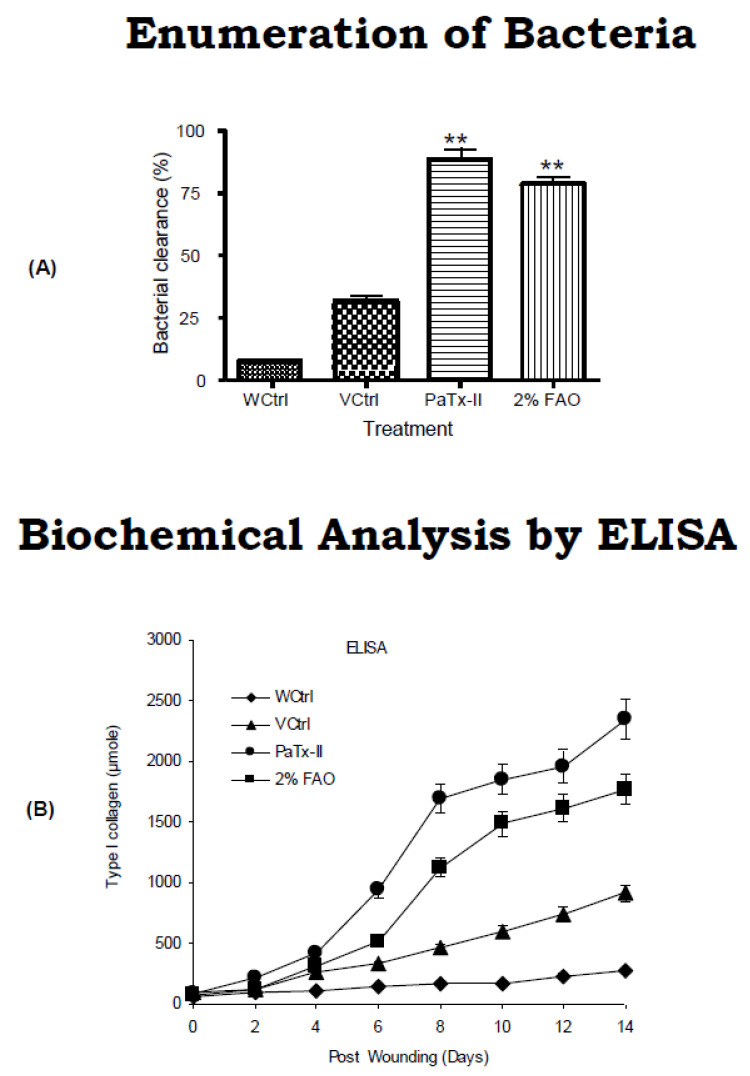
Wound fluid bacterial killing assay based on colony forming units (CFU/mL). (**A**) PaTx-II effectively inhibited the growth of *S. aureus* and completely cleared the growth of bacteria after 14 days versus the control. Graphical representation of wound fluid (WF) shows the percentage inhibition of bactericidal clearance after 24 h incubation at 37 °C. (**B**) Collagen accumulation and breaking strength of wounds were compared. New collagen accumulation was found in excised wounds and control skin. The difference between the curves was calculated, representing collagen accumulation. Statistical comparisons were performed with Prism 4.0 Software (Graph-Pad), using a two-tailed Student’s *t*-test for the comparison of two data sets, and ANOVA for multiple comparisons. Statistically significant differences from controls were observed for PaTx-II and FAO (*p* < 0.01; **).

The efficacy of PaTx-II in clearing *S. aureus* was confirmed by the results of the analysis of wound fluid (WF). Bacterial replication was not observed following treatment with 2% FAO, and a greater than 98% reduction in bacterial skin counts was observed in PaTx-II-treated mice 14 days after treatment, as compared with untreated controls (Figure 6A).

### 2.8. Estimation of Collagen by ELISA

The role of collagen in wound healing during the first two days, and rates of new collagen deposition for excised wounds and skin controls were the same (Figure 6B). However, the rate of collagen synthesis and deposition in wounds increased significantly (*p* < 0.01) between 6 and 14 days post-wound. Healing of wounds with collagen synthesis and deposition continued at a rapid rate after 14 days. There was a marked increase of collagen levels in the PaTx-II-treated wounds and 2% FAO-treated mice, and there was a strong decrease in inflammatory cells. COX-2 and TNF-α were strongly associated with inflammatory cells, in which these biopsies were present in the superficial layers of the wound controls (WCtrl and VCrtl).

### 2.9. Western Blots of PaTx-II for Wound Healing

**Figure 7:**
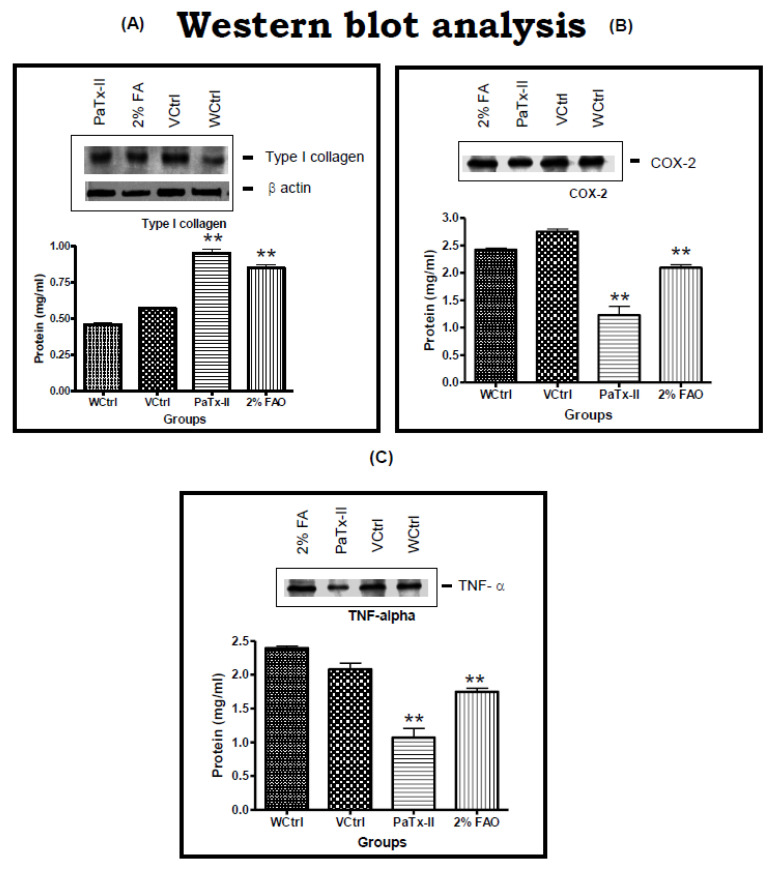

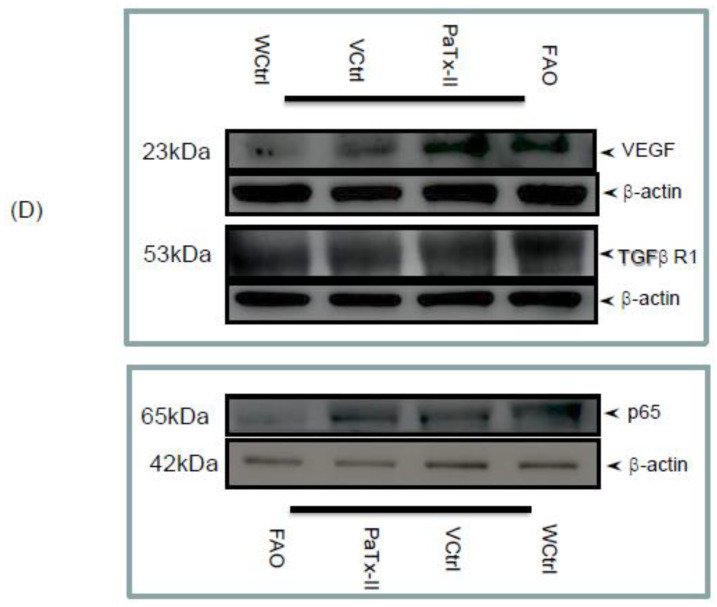
Western blot analysis of the protein extracted from treated infected wound/control tissues. (**A**) Local application of PaTx-II (0.5 mg/kg, bw) or FAO shows a significant up-regulation of collagen I at 14 days compared to controls. (**B**) Effects of PaTx-II or FAO on COX-2 protein levels. A significant decrease in COX-2 activity at day 14 was observed for both at day 14 relative to the control (WCtrl and VCtrl) groups. (**C**) In the wound assay control mice, TNF-α was up-regulated after injury, whereas expression was significantly decreased after mice were treated with PaTx-II or FAO. ** *p* < 0.01. (**D**) Western blot analyses shows a significant up-regulation of vascular endothelial growth factor (VEGF) and transforming growth factor β (TGF-β) influenced faster neovascularization after the topical application of PaTx-II (0.5 mg/kg, bw) at 14 days compared to the control.

Alteration of collagen I expression in wounds was quantified by Western blot within the treated and control animals: WCtrl wound alone, VCtrl control, and infected wound treated with 0.5 mg/kg/day neutral gel, PaTx-II (0.5 mg/kg, b.w.) and 2% FAO (0.5 mg/kg, b.w.). Collagen I antibody formed a single band at a molecular mass identical to the positive collagen control (Figure 7A). Both PaTx-II-treated mice and 2% FAO-treated mice show a darker band for collagen I compared to the control mice. Untreated and infected wounds have higher levels of inflammatory markers (TNF-α, COX-2) and phosphor p65 14 days post-wound, as observed by Western blot analysis (Figure 7B,C). There was a significant up-regulation of vascular endothelial growth factor (VEGF), and transforming growth factor β (TGF-β) influenced faster neovascularization after the topical application of PaTx-II (Figure 7D).

### 2.10. Proinflammatory Cytokines in Wound Healing

**Figure 8:**
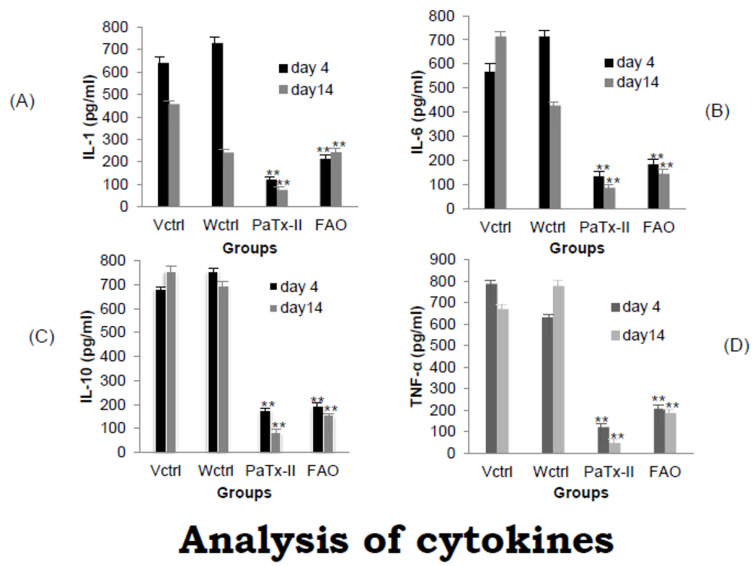
Proinflammatory cytokines were analyzed by an ELISA assay before and after wound treatment in mice. (**A**–**D**) There were increased levels of the cytokines IL-1, IL-6, IL-10 and TNF-α in untreated, infected wound-control mice, whereas both PaTx and FAO-treated mice show significantly decreased levels of all 4 cytokines after 4 and 14 days of treatment. For treated wounds, the PaTx-II-treated mice show a greater reduction of inflammatory cytokines after 4 and 14 days of treatment compared to the 2% FAO-treated mice. ** *p* < 0.01.

Levels of the proinflammatory cytokines interleukin-1 (IL-1), IL-6, IL-10, and TNF-α were analyzed by ELISA over the course of wound healing. IL-1 and TNF-α play an important part in the commencement of inflammation and are essentially required for wound transformation and the reduction of inflammation (Figure 8A–D). Elevated levels of the cytokines IL-1, IL-6, IL-10, and TNF-α were observed in untreated mice wounds, whereas the PaTx-II or 2% FAO-treated mice show a significantly greater reduction of inflammatory cytokines after 14 days of treatment. This reduction was also greater in PaTx-II-treated mice than in 2% FAO-treated mice. These results suggest that inflammatory cytokines may be associated with controlling the wound healing process; however, their decrease in treatment groups may also simply be associated with a lower degree of bacterial burden.

## 3. Discussion

The potential of snake toxins as antibiotics has previously been recognized in a cluster of Lys49 PLA_2_ homologs found in snake venom with bactericidal activity [30]. In the present work, we report the isolation of the novel antimicrobial PaTx-II, which possesses the potential therapeutic application against *S. aureus*, as compared to other PLA_2_s, including PaTx-I, which is also described. In order to identify how PaTx-II inhibits *S. aureus*, we probed the mechanism of action, which included morphological alterations, by scanning and transmission electron microscopies. PaTx-II-induced pores on the bacterial cell wall and caused cell lysis, similar to changes in the cell wall/membrane integrity observed when *S. aureus* is exposed to chloramphenicol or fusidic acid [49]. Thus, mechanisms by which PaTx-II inhibits *S. aureus* appear to differ from those reported for neutrophil α-defensins, where lamellar mesosome-like structures occur at the cell wall [50].

Snake venom PLA_2_s, metalloproteases, and hyaluronidases were commonly reported with venom toxicity, and most antimicrobial enzymes/proteins derived from the venom display cytotoxic effects on eukaryotic cells [51]. To address the lack of specificity, we assayed the in vitro cytotoxicity of PaTx-II on human skin (HEPK), lung fibroblast (MRC-5), and macrophage (THP-1) cells. PaTx-II was unable to induce cell death or lytic effects at the highest concentration (CC_50_ > 1000 µM), and no morphological changes were observed in light micrographs. Our findings of no toxicity were similar to the recently reported cardiotoxin 1 (CTX-1)-derived peptides NCP-2/NCP-3 tested against MDBK cells; both peptides showed low toxicity toward non-target eukaryotic cells (cell death less than 10% at 100 µg/mL concentrations) [39].

In the murine skin infection wound healing model, PaTx-II-treated animals show complete healing after approximately two weeks. Bacterial growth inhibition was obtained after 14 days for the different treatment groups: more than 90% for the PaTx-II-treated animals, 83% for 2% FAO-treated animals, and only 33% for VCtrl and 8% for WCtrl. Bacterial growth was abundant on day four but fully cleared after 14 days upon treatment with PaTx-II. Histological examination of *S. aureus*-induced skin lesions from control (WCtrl and VCtrl) mice show abundant neutrophilic infiltrates compared to those observed in the PaTx-II-treated mice. Dermal wound healing involves a sequence of events, such as tissue transformation, comprising an inflammatory reaction [52]. This progression seems to be arbitrated by the soluble cytokines and growth factors that affect various cell types, such as keratinocytes, dermal fibroblasts, and vascular cells [53].

MT stain was used to analyze the expression of collagen in wounds. The PaTx-II-treated mice show significant collagen expression; however, there was no detectable collagen expression in the WCtrl and VCtrl groups. These results suggest that, in addition to affecting collagen expression, the influx of inflammatory cells also inhibited collagen deposition in the wound [54]. Collagen activity and subsequent deposition were the main aspects of tissue repair and wound healing. In our study, ELISA results indicate higher levels of type I collagen in healed wound tissues after PaTx-II treatment versus controls. Our results also suggest that mice fibroblasts were richly expressing collagen I after treatment with PaTx-II, which was considered an essential hallmark of wound healing [55,56]. In the present study, intense immunostaining of type-I collagen was strongly associated with regions containing high granules, as previously observed [57]. The latter reported that the transcriptional activity of type I collagen gene expression was controlled by TNF-α and TGF-β1 [58,59,60].

Levels of the proinflammatory cytokines IL-1, IL-6, and TNF-α were elevated during wound healing, consistent with previously reported results showing elevated IL-1, IL-6, IL-10, TNF-α, IL-2, IL-8, IL-13 and granulocyte colony-stimulating factor (G-CSF) in the skin infection wound model [61,62]. Elevated levels of these cytokines were also found clinically in burn patients’ acute phase reactions and distinct inflammatory responses [62]. In addition, there was an elevated expression of IL-1 in wounded murine models (thermal injury) and human skin [63]. IL-1 triggers the migration of inflammatory cells and keratinocytes in vivo [64], and IL-1 induces fibroblast proliferation and the production of fibronectin, collagen, matrix metalloproteases (MMPs), and tissue inhibitors of metalloproteases [65,66,67]. In the present study, Western blots were evaluated in the presence of collagen I, TNF-α, and COX-2 in murine skin wound models. In controls, COX-2 was expressed at significant levels in various wounded tissues, such as the epidermis, with inflammation and cell activation by various agonists that include cytokines and growth factors [68]. In contrast, the PaTx-II-treated mice show more significant amounts of collagen I synthesis after two weeks of treatment than controls or the 2% FAO-treated mice. TNF-α expression levels were also significantly enhanced 14 days after injury. These results suggest that inflammatory cytokines may control the wound healing process. Conversely, untreated mice exhibit an increased expression of the inflammatory markers TNF-α and COX-2 in wounds, which likely interfere with wound repair [69,70,71].

The nuclear transcription factor kappa B (NF-kB) signaling pathway was regulated and complicated regarding the pathogenesis involving many inflammatory disorders [17]. Previous reports showed that NF-kB phospho-p65 staining and inflammatory cells were more evident in null wounded mice compared to wildtype [72]. P65 protein translocates into the nucleus and also induces the expression of proinflammatory genes such as TNF-α, IL-1β, and p65, which activate NF-kB signaling [17].

In summary, PaTx-II exerted strong antimicrobial activity with severe membrane damaging effects on *S. aureus* in an in vitro system. It is also very evident that good in vivo efficacy is obtained when it is topically applied in a mouse model infected by *S. aureus*, exhibiting wound healing potential. As small protein and peptide synthesis are relatively easier, this finding will open up the possibility of manipulating tailor-made peptides derived from the PaTx-II parent protein, which could be helpful in veterinary or therapeutic applications.

## 4. Materials and Methods

### 4.1. Venom

Lyophilized crude venom of *P. australis* (Australian king brown snake) was obtained from Venom Supplies Pte Ltd., Tanunda, South Australia.

### 4.2. Protein Assay

Isolated protein was prepared at 4.0 mg/mL doses, with bovine serum albumin (BSA) used to establish a standard curve. Protein concentrations were estimated by the standard protocol of [73], as adapted by BioRad Laboratories (San Diego, CA, USA).

### 4.3. Isolation of PaTx-II from Venom

Venom (2 gm) was dissolved in 20 mL of 50 mM Tris-hydrochloric acid buffer, pH 7.4 (Tris-HCL), and subsequently centrifuged at 500× *g* at 4 °C for 15 min. Clear venom supernatant was applied to a Superdex G-75 column (1.6 × 40 cm; Amersham Pharmacia, Sweden) using 50 mM Tris-HCL buffer and a flow rate of 1.5 mL/min. All collected fractions were monitored at 280 nm and six peaks (Pa1–Pa6) were tested for biochemical activities and protein content. The fractions (Pa4) containing antibacterial and PLA_2_ activities were further isolated by reverse-phase (RP)-HPLC on a C18 column (250 × 4.60 mm, 5 microns, 100 Å, Jupiter Phenomenex, Sweden) using a flow rate of 1 mL/min (0.1% aqueous trifluoroacetic acid) (TFA, Sigma Aldrich St Louis, MO, USA). A gradient of 0.1% water in TFA with a linear gradient to acetonitrile 75% B over 70 min was subsequently resolved into six peaks (Pa-F1 to Pa-F6). The most active peak (Pa–F2) was further purified by HPLC using a C8 column in 0.1% water in TFA with a linear gradient to 80% acetonitrile (Merck, Germany) in 0.1% TFA and a flow rate of 1 mL/min for 60 min. Protein absorbance was monitored at 280 and 254 nm [74], and this step yielded the isolated protein PaTx-II.

### 4.4. MALDI-TOF Spectrometry

The fraction containing PaTx-II was desalted and analyzed by MALDI-TOF/MS (Perspective Biosystems, Voyager-DE mass spectrometer, Framingham, MA). Edman degradation analyzed the N-terminal sequence of the *P. australis* PLA_2_ using Applied Biosystems, 494 pulsed liquid-phase sequencers equipped with an online 120A PTH-amino acid analyzer. Protein sequences were analyzed by the search engine, Basic Local Alignment Search Tool (BLAST), and retrieved sequences were aligned using CLUSTAL W 1.82.

### 4.5. PLA_2_ Enzyme Activity

The PLA_2_ activity of the PaTx-II enzyme was determined by the Cayman Chemical secretory PLA_2_ (sPLA_2_) assay kit that uses diheptanoyl phosphatidylcholine as a substrate. Upon hydrolysis of the thioester bond at the *sn*-2 position by PLA_2_, free fatty acid thiols are identified with 5,5-dithio-bis-2-nitrobenzoic acid (DTNB) via an increase in absorbance at 405 nm, using the manufacturer’s instructions [75].

### 4.6. In Vitro Antimicrobial Assays

Antimicrobial susceptibility was evaluated by a disc-diffusion method [40,76], and minimum inhibitory concentrations (MICs) were assayed by the broth-dilution method following the National Committee for Clinical Laboratory Standards (NCCLS) [77]. Fresh overnight bacterial cultures of *Escherichia coli*, *Enterobacter aerogenes*, *Proteus vulgaris*, *Proteus mirabilis*, *Pseudomonas aeruginosa*, and *Staphylococcus aureus* were obtained from the Department of Microbiology, NUS, Singapore. Cultures were suspended to a turbidity of 0.5–1.2 McFarland units. Bacterial solutions were diluted by Mueller Hinton (MH) broth (Oxoid, UK) and incubated for five hours to obtain an exponential phase. The bacterial suspensions were introduced (0.5 × 10^5^–1.2 × 10^6^ colony forming units, CFU/mL) into wells having serial dilutions of PaTx-II at final concentrations of 140, 120, 100, 80, 60, 40, and 20 µM in 1 × phosphate buffer saline (PBS, pH 7.4) in plates with MH broth. Control plates contained bacteria with normal physiological PBS added. The antibiotics chloramphenicol and fusidic acid (FAO, 30 µg/disc) were used as a reference control. Adjusted inoculums of 1 × 10^6^ CFU/mL were introduced into every well with 50 µL of PaTx-II. The 96-well plates were incubated at 37 °C for 24 h, and inhibition of bacterial growth was assessed by quantifying the absorbance at 560 nm (Benchmark plus Spectrophotometer, BioRad, USA). Experiments are reported as the mean values of three independent determinations (n = 3).

### 4.7. Cytotoxic Effect of PaTx-II on Human Cells

#### 4.7.1. Cell Culture

Skin (HEPK)/lung (MRC-5) fibroblasts, human leukemia monocytic (THP-1) macrophage cells were purchased from American Type Culture Collection (ATCC, Manassas, Virginia, USA). Roswell Park Memorial Institute (RPMI) 1640 cell culture media and fetal bovine serum (FBS) were obtained from Gibco (5505 Veterans Blvd, Pascagoula, Mississippi, USA). Polylysine, MTT [3-(4,5-dimethylthiazol-2-yl)-2,5-diphenyl tetrazolium bromide] and PBS were obtained from Sigma-Aldrich, St. Louis, MO, USA.

#### 4.7.2. Cell Viability by MTT Assay

The 2.5-diphenyl-2H-tetrazolium bromide (MTT) assay was used to measure cellular metabolic activity as an indicator of cell viability, proliferation, and cytotoxicity. Skin (HEPK)/lung (MRC-5) fibroblasts, human leukemia monocytic (THP-1) macrophage cells were cultured in a 5% CO_2_ incubator at 37 °C for 24 h in culture media containing 10% fetal calf serum, 100 U/mL penicillin, and 100 U/mL streptomycin in 96 well-micro titer plates. HEPK cells were incubated in the presence of PaTx-II (0.01–1000 µM) for 24 and 48 h. Cells without toxin exposure served as a control. Then, 20 µL of MTT reagent (5 mg/mL in 1 × PBS) was introduced into each well and incubated 4 h at 37 °C. The optical density (OD) values were recorded at 490 nm by using an ELISA plate reader [78].

### 4.8. Ethics Approval

Animal experiments were performed in accordance with the protocol-specific procedure approved by the National University of Singapore (NUS), Institutional Animal Care and Use Committee (IACUC), which oversees the care and use of test animals for research purposes.

### 4.9. Mouse Model of Skin Infection

A mouse model for skin wounds was created by modifying a previously reported infection model [41,79,80,81]. Experimental mice were divided into four groups (n = 5 each). The backs of 8-week-old BALB/c female mice (25–30 g) were shaved, and after injection of anesthesia (0.2 mL of Ketamine + Medetomidine, intra-peritoneally (i.p)), a wound (5 mm x 4 mm, 20 mm^2^) was created in the skin on day 0. Then, 50 µL of a mid-logarithmic growth phase of *S. aureus* (5 × 10^7^ CFU/mL) was applied topically to the wound area and left for two days to maximize wound establishment. Group I (sterile PBS only) served as a wound treatment control (WCtrl); Group II received an aqueous gel used as a vehicle control VCtrl). Group III was treated with PaTx-II (0.5 mg/kg, body weight) mixed with gel, and Group IV was treated with 2% fusidic acid ointment (0.5 mg/kg, b.w.), or Fucidin (Sigma Aldrich, St Louis, MO, USA) as a reference drug. The protein (PaTx-II) and the drug fusidic acid ointment (FAO) was applied for four days (twice a day) to the wound and repeated throughout the experiment thrice (n = 3). Wound closure was checked, and the size of the wound was recorded every day until day 14. At the experiment’s endpoint (14 days), the animals were sacrificed, and the tissue was harvested. The infected area of the skin was stored for histology and biochemical analysis. Statistical significance of wound data within the groups was analyzed by one-way ANOVA and a Student’s *t*-test. Reductions of wound areas are represented as a percentage of the wound closure.

### 4.10. Bacterial Count Wound Assay

Counts of bacteria were determined from the control and PaTx-II-treated samples. Wound samples were homogenized by an electric homogenizer (Omni International, Kennesaw, Georgia, USA) and suspended in 2 mL of normal physiological saline. Suspensions were inoculated in specific dilutions on Mueller Hinton (MH) agar (Oxoid, Basingstoke, UK) and incubated at 37 °C for 24 h. Bacterial loads were calculated as the number of *S. aureus* colony-forming units per gram (cfu/g) of tissue [79].

### 4.11. Histological Examination

Mice were sacrificed by CO_2_ inhalation and wound tissue was harvested (~6 mm) from treated and controlled wounds at selected time points (2–14 days). Wound tissues were fixed in 10% formalin (Fisher Scientific, NJ, USA), processed for dehydration, embedded in paraffin (Paraplast^TM^ resin), and cut in half. The embedded tissues were laid flat and cut with a single-edge razor blade placed vertically over the middle of the wound. Thin sections (8 µm) were stained with hematoxylin and eosin (H&E) [82,83]. For pathological evaluation, the sections were examined under a light microscope (Olympus, USA).

### 4.12. Masson’s Trichrome (MT) Stain for Collagen

The MT staining method revealed skin collagen in the treated wound and control groups. After de-paraffinization, all skin sections were stained with Weigert’s hematoxylin for 3 min, washed in water for 3 min, and stained in acid fuchsin solution for 5 min. The sections were rinsed in water for 3 min. All sections were treated with phosphomolybdic acid solution for 5 min and, after draining completely, were stained with methyl blue solution for 5 min and rinsed in water. The sections were treated with 1% acetic acid for 2 min and dehydrated in an alcohol sequence (50–100%). Slides were examined for collagen under a light microscope [84].

### 4.13. Immunofluorescence Staining

Immunolocalization was performed in the following groups: Group (I) WCtrl (wound control), Group (II) VCtrl (vehicle control), Group (III) infected wound (received with 0.5 mg/kg) PaTx-II, Group (IV) (positive control), and 2% fusidic acid ointment (0.5 mg/kg) treated wound as a standard. Mice were sacrificed with excess exposure of carbon dioxide (CO_2_) inhalation and wound tissues fixed in 30% Bouins fluid. Paraffin sections (7 μm) of groups were incubated overnight at 4 °C with antibodies for type I collagen (PanCytokeratin), tumor necrosis factor-alpha (TNF-α), or cyclooxygenase-2 (COX-2) at a dilution of 1:500 in PBS with 1% bovine serum albumin (BSA) (Santa Cruz Biotechnology Inc, Santa Cruz, California, USA), followed by 1 h incubation at 28 °C. The secondary antibody (polyclonal anti-rabbit IgG HRP) was incubated at 1:5000 dilution in PBS with 12% BSA. Subsequently, sections were washed in PBS containing 0.1% Tween-20, before mounting with 0.5 µg/mL of DAPI stain (Sigma, St Louis, Missouri, USA) and photographed on an Olympus fluorescence microscope [85]. Sectioning with 3,3-diaminobenzidine (DAB) staining was also performed for collagen (type I).

### 4.14. Western Blot Analysis of PaTx-II for Wound Healing

Proteins were separated by 10-12% sodium dodecyl sulfate-polyacrylamide gel electrophoresis (SDS-PAGE) as previously reported by [86] and transferred to the nitrocellulose membrane. The membrane was blocked in 5% powdered milk in TBS-T (10 mM Tris-HCl, pH 8.0, 150 mM NaCl, 0.05% Tween 20) and subsequently incubated with primary antibodies against type I collagen, COX-2, TNF-α, or beta-actin (Santa Cruz, CA, USA). These antibodies were incubated overnight at 1:1000 dilutions (4 °C), membranes were probed with anti-mouse IgG-HRP conjugated polyclonal secondary antibody, incubated for 1 h at 1:5000/1:10,000 dilutions (Santa Cruz, CA, USA), and proteins were visualized by the ChemiDoc Imaging System (Bio-Rad, Hercules, CA, USA).

### 4.15. Biochemical Analysis of Collagen (Type I)

Wound tissues were excised and homogenized with 300 µL of 5% trichloroacetic acid (TCA) (Sigma Co., St Louis, MO, USA) or tissue extraction (TE) lysis buffer containing a mixture of 10 mM PBS, 0.1% SDS, and 5 mM EDTA (Roche Diagnostics, Tokyo, Japan). Tissue suspensions were centrifuged at 15,000 rpm for 15 min, and the supernatant was used for an enzyme-linked immunosorbent assay (ELISA). Type I collagen, TNF-α, and COX-2 proteins were estimated with commercial ELISA kits (BioSource, Inc., Camarillo, CA, USA) according to the manufacturers’ recommendations. The total protein content of the tissue supernatant was determined with a commercial kit (BCA Protein Assay Kit, Pierce, Rockford, IL) using Bovine Serum Albumin (BSA) as a standard. The data were analyzed as collagen (pg/mL)/total protein (mg/mL) for each sample [87].

### 4.16. Measurement of Proinflammatory Cytokines in Wound Healing

We studied the proinflammatory cytokine responses in wounds before and after treatment. Wound tissue samples (50 mg) were homogenized on ice with an electric grinder in 1 mg/mL Tris buffer (50 mM Tris, pH 7.4) containing protease inhibitors; the homogenate was then centrifuged at 2500 g for 10 min at 4 °C. The clear wound fluid was prepared and stored at −80°C for cytokine assays. Wound fluid was diluted (1:1) with the sample buffer. The levels of cytokine (IL-1β, IL-6, IL-10, and TNF-α) were analyzed by an ELISA assay at 450 nm. The cytokines were measured in the tissue samples (pg/mL) relative to total protein levels in the sample. The total protein was estimated using the BCA assay (Bio-Rad, Hercules, CA, USA).

### 4.17. Statistical Analysis

The data were analyzed as mean ± standard deviation (S.D.) of five biological replicates (n = 5) and the coefficient of variation was calculated through one-way ANOVA. The student’s *t*-test analyzed statistical significance. The values of * *p* < 0.05 and ** *p* < 0.01 were considered statistically significant compared to the control.

## Figures and Tables

**Table 1:** Phopholipase A_2_ (PLA_2_) activities determined from the *Pseudechis australis* (king brown snake), in vitro bacterial screening of crude venom, purified fractions and proteins by standard procedures.

Fraction	Protein (mg)	PLA_2_ Activity (nmoles/Unit of Enzyme)	*S. aureus*	*E. aerogenes*	*P. vulgaris*	*P. mirabilis*	*E. coli*	*P. aeruginosa*
Pa-crude venom	2000	1028	+++	++	+	+	-	-
Superdex G-75								
Pa1	149.5	1.3	-	-	-	-	-	-
Pa2	312	177.8	+	+	-	-	-	-
Pa3	213	121	+	+	-	-	-	-
Pa4	962	1134	+++	+++	+	+	-	-
Pa5	112	18.9	-	-	-	-	-	-
Pa6	202	11.2	-	-	-	-	-	-
Sepharose C18								
Pa–F1	728	268.2	+	+	-	-	-	-
Pa–F2	821	1225	++++	+++	+	+	-	-
Pa–F3	12.9	124.9	-	-	-	-	-	-
Pa–F4	104	128.1	-	-	-	-	-	-
Pa–F5	5.3	174.1	-	-	-	-	-	-
Pa–F6	6.6	142.0	-	-	-	-	-	-
Sepharose C8								
PaTx-I	325	289.1	+	-	-	-	-	-
PaTx-II	996.3	1283.1	++++	+++	++	++	-	-
Chloramphenicol	-	-	++++	+++	+++	+++	++	++
Fusidic acid	-	-	++++	+++	++	++	++	++

Protein (PaTx-II) inhibits bacteria and forms a clear zone of inhibition around the discs. Results are shown as grading of inhibition zone: - (no activity), (+) 7 mm in diameter (mm), ++ (10–15 mm), +++ (15–20 mm), and ++++ (20–35 mm). Bacteria used in this assay include *Staphylococcus aureus*, *Enterobacter aerogenes*, *Proteus vulgaris*, *Proteus mirabilis*, *Escherichia coli*, and *Pseudomonas aeruginosa*. PaTx-I failed to show any effects against the tested bacteria, including *Pseudomonas aeruginosa*, etc.

## Data Availability

Not applicable.

## Abbreviations

AVA	Agri-Food and Veterinary Authority of Singapore
CFU	Colony forming units
COX-2	Cyclooxygenase-2
DAPI	4′-6-diamiinol-2-phenylindole
FAO	Fusidic acid ointment
ELISA	Enzyme-linked immunosorbent assay
IL-1β	Interleukin-1beta
IACUC	Institutional Animal Care and Use Committee
MRSA	Methicillin-resistant *Staphylococcus aureus*
MT	Masson’s trichome staining
MMPs	Metalloproteases
MAP	Mitogen-activated protein kinases
NACLAR	National Advisory Committee for laboratory Animal Research
PaTx-II	*Pseudechis australis* toxin-II
RP-HPLC	Reverse-phase high-pressure liquid chromatography
svPLA_2_	Snake venoms phospholipases A_2_
sPLA_2_	Secretary phospholipases A_2_
SAP	Serum amyloid protein
Tris-HCL	Tris-hydrochloric acid buffer
TFA	Trifluoroacetic acid
TNF-α	Tumor necrosis factor-alpha
TE	Tissue extraction lysis buffer
VCtrl	Vehicle control
WCtrl	Wound control
NV	Neovascularization
